# Supplementary material for: Impact of prophylactic oral azithromycin during labor on Azithromycin Resistance (AMR) in nasal Staphylococcus aureus and Streptococcus pneumoniae in women and infants in the multi-country Azithromycin Prevention in Labor Use Study (A-PLUS)
Source: PLoS One. 2026 Apr 17;21(4):e0346174. doi: 10.1371/journal.pone.0346174 (PMC13089724; doi:10.1371/journal.pone.0346174)
Supplement: S1 Table — (DOCX) [file pone.0346174.s001.docx]

**Supplementary Table 1: Nasal Specimens Collected in the AMR Sub-Study Over Time**

| **Time Point** | **Women** | | | | **Infants** | | | |
| --- | --- | --- | --- | --- | --- | --- | --- | --- |
|  | **Azithromycin (n=459)** | | **Placebo**  **(N=451)** | | **Azithromycin**  **(N=457)** | | **Placebo**  **(N=451)** | |
|  | **N** | **% (Of women enrolled)** | **N** | **% (Of women enrolled)** | **N** | **% (Of infants enrolled)** | **N** | **% (Of infants enrolled)** |
| Baseline | 459 | 100 | 450 | 100 | 454 | 99 | 444 | 98 |
| Day 7 | 411 | 90 | 412 | 91 | 405 | 89 | 408 | 91 |
| 6 weeks | 374 | 82 | 380 | 84 | 368 | 81 | 380 | 84 |
| Month 3 | 328 | 72 | 340 | 75 | 325 | 71 | 335 | 74 |
| Month 6 | 327 | 71 | 311 | 69 | 321 | 70 | 305 | 68 |
| Month 12 | 333 | 73 | 334 | 74 | 325 | 71 | 327 | 73 |
| Overall |  | 81 |  | 82 |  | 80 |  | 81 |
