# Supplementary material for: Impact of prophylactic oral azithromycin during labor on Azithromycin Resistance (AMR) in nasal Staphylococcus aureus and Streptococcus pneumoniae in women and infants in the multi-country Azithromycin Prevention in Labor Use Study (A-PLUS)
Source: PLoS One. 2026 Apr 17;21(4):e0346174. doi: 10.1371/journal.pone.0346174 (PMC13089724; doi:10.1371/journal.pone.0346174)
Supplement: S2 Table — (DOCX) [file pone.0346174.s002.docx]

**Supplementary Table 2: Nasal Carriage of *S. aureus* and *S. pneumoniae* in the AMR Sub-Study**

| **Time Point** | ***S. aureus*** | | | | ***S. pneumoniae*** | | | |
| --- | --- | --- | --- | --- | --- | --- | --- | --- |
|  | **Women** | | **Infants** | | **Women** | | **Infants** | |
|  | **Azithromycin %** | **Placebo %** | **Azithromycin %** | **Placebo %** | **Azithromycin %** | **Placebo %** | **Azithromycin %** | **Placebo %** |
| Baseline | 41.4 | 40.2 | 31.7 | 30.6 | 18.1 | 16.4 | 17.0 | 15.5 |
| Day 7 | 41.4 | 46.6 | 46.4 | 47.8 | 18.0 | 15.8 | 17.5 | 15.7 |
| 6 weeks | 41.7 | 49.7 | 40.5 | 45.0 | 17.6 | 17.1 | 18.8 | 17.1 |
| Month 3 | 38.4 | 43.2 | 35.7 | 35.8 | 17.1 | 15.0 | 19.1 | 20.9 |
| Month 6 | 33.6 | 35.0 | 24.9 | 26.6 | 16.5 | 14.5 | 23.1 | 23.3 |
| Month 12 | 32.4 | 31.1 | 27.1 | 28.7 | 16.2 | 15.0 | 23.1 | 20.8 |
| Overall | 38.5 | 41.4 | 34.8 | 36.2 | 17.3 | 15.7 | 19.5 | 18.5 |
