# Supplementary material for: Impact of prophylactic oral azithromycin during labor on Azithromycin Resistance (AMR) in nasal Staphylococcus aureus and Streptococcus pneumoniae in women and infants in the multi-country Azithromycin Prevention in Labor Use Study (A-PLUS)
Source: PLoS One. 2026 Apr 17;21(4):e0346174. doi: 10.1371/journal.pone.0346174 (PMC13089724; doi:10.1371/journal.pone.0346174)
Supplement: Attachment — Submitted filename: 2 Rebuttal Letter.docx [file pone.0346174.s003.docx]

**Reviewer #1: As the statistical reviewer I will focus on methods and reporting.

Major**

**Comment #1:** Please discuss that the recruitment target was not met as a limitation in the relevant section, which has power implications.

**Response**: Thank you for this comment, we have updated the text in limitations in order to address this issue as a limitation.

**Comment #2:** the focal point of the analysis should be the logistic regression model. if the model allows I would be keen to include a random intercept for site. I am not sure the interaction is needed, I would suggest removing it if it is found not to add anything, to simplify the model.

**Response**: The logistic regression model was used solely to compare estimates between Africa and Asia. The primary analysis was the Cochran-Mantel-Haenszel test stratified by region, which was used to test for association between resistance and treatment. To clarify this, we have updated the text regarding the statistical analysis to make it clearer.

**Comment #3:** there is no information in the methods section as to how the authors dealt with missing data. why weren't multiple imputation approaches considered?

**Response**: No differences in baseline characteristics were noted between mothers and infants with and without a nasal swab over time so we chose to do a complete case analysis at each time point. We have added a line in the methods to emphasize this.

**Comment #4:** I would move away for all p-value focused analyses, since it is impossible to evaluate p-values as descriptive. they are either adjusted and informative or not. Please move all analyses to a regression framework and focus reporting effect/association sizes and their confidence intervals, still keeping the explanatory label.

**Response**: Our objective was just to test association between arm and resistance controlling for region, our analysis was not an attempt to get estimates of association. We have adjusted the text to make the intended statistical analysis clearer.

**Minor**

**Comment #1:** the authors mention 125 women per site before they explain the power calculations. please mention the power calculation alongside the recruitment targets per site.

**Response**: We have moved the sample size discussion to before we mention the 125 women per site in order to clarify the sample upfront.

Reviewer #2:

This is an important and timely manuscript reporting antimicrobial resistance (AMR) outcomes from the multicountry A-PLUS randomized controlled trial. The study examines azithromycin resistance among nasal Staphylococcus aureus and Streptococcus pneumoniae isolates from mothers and infants following intrapartum azithromycin administration across seven low- and middle-income countries.
The work is scientifically rigorous, methodologically sound, and highly relevant to global efforts to balance the benefits of prophylactic antibiotic interventions with their AMR risks. The multicountry scope, standardized methods, and longitudinal sampling represent major strengths.
However, certain aspects of clarity, methodological detail, and interpretative depth should be improved before publication.

**Major Comments

Comment #1:** The introduction outlines three AMR assessments (nasal, rectal, genomic), but this paper focuses only on nasal carriage. In the last paragraph, specify that this manuscript reports findings from the nasal *S. aureus* and *S. pneumoniae* analysis.

**Response:** Thank you for this comment. This change has been made in the last paragraph of the introduction.

**Comment #2:** The culture yield for *S. pneumoniae* is low, with over 90% undetermined samples at several time points. This raises concerns about representativeness and statistical power. Discuss potential reasons for example sample transport, viability, timing, etc and possible implications for bias in prevalence estimates.

**Response**: We agree that low culture yield for *S. pneumoniae* is a concern and is a study limitation. We have added 3 sentences to the Conclusions/Discussion section addressing this, particularly highlighting difficulty in culturing this bacteria as well as small variations in collection, transport, and laboratory methods that can result in no viable recovery. We also note that all laboratory staff were trained in the same standard operating procedures and used the same recommended consumables. We also used ongoing quality control and validation procedures for all sties as described in the methods and results.

**Comment #3:** Baseline resistance rates are not negligible and may reflect pre-existing macrolide exposure. Expand discussion on background community azithromycin use and acknowledge its potential confounding effect.

**Response**:

We agree with this concern and have added more detail in the first paragraph of the Discussion about use of azithromycin for treatment of COVID, particularly in the Asian sites.

Minor Comments

**Comment #1:** Consider revising the first line of the abstract line 65 - 66 emphasizing the balance between mortality/sepsis prevention and AMR risk. for example .. Prophylactic intrapartum azithromycin reduces maternal mortality/sepsis but may promote antimicrobial resistance (AMR) in commensal bacteria.

**Response:** Thank you for this suggestion and we have made this change as suggested.

**Comment #2:** Aside from mothers giving consent where there any other inclusion criteria to join for the AMR sub study

**Response:** We have added that there were no additional eligibility criteria for women selected for the AMR sub-study other than willing ness to participate and signing of the AMR sub-study consent form.

**Comment #3:** were the laboratory team blinded to treatment allocation?

**Response:** The laboratory staff were blinded to treatment allocation and we have updated the text to address.

**Comment #4:** What was the concentration of azithromycin in azithromycin containing agar used to select organisms for identification and susceptibility testing?

**Response**: The concentration of azithromycin added to the agar was dependent on the bacterial species and determined based on the recommended MIC breakpoints for resistance (CLSI , 2024 – reference 13).

**Comment #5:** what does resistant undetermined mean?

**Response:** Thank you for noticing that there are minor discrepancies in the text when resistance undetermined is mentioned. We have reviewed all instances of “resistance undetermined” and now the consistently are clarified as “because the cultures failed to grow or had incomplete laboratory testing”.

**Comment #6:** Highlight that the rebound to baseline resistance levels by 6–12 months aligns with previous evidence from The Gambia and Kenya.

**Response:** We already have indicated that our results are similar to the Gambia study in paragraph 3 of the conclusions/discussion. We have clarified that the Gambian results returned to baseline in paragraph 3 in the conclusions/discussion section. I think the Kenyan study refers to our Kenya site so that is already included in our date.

**Comment #7:** Be consistent in the use of American or UK English for example labor or labour.

**Response:** We did a search for English vs. US spellings and noted that all mentions of “labour” were in the references (8, 10 and 20). We believe that there are no outstanding instances to UK English references other than in the references, which we hesitate to change. Similarly, we have not changed institution names that use the English spelling.

**Comment #8:** Where possible, provide absolute prevalence percentages in-text when comparing treatment arms (e.g., “Azithromycin-resistant S. aureus was X% vs. Y% at 6 weeks”).

**Response:** We have provided this information in the text (RESULTS under Section “Cross sectional Analyses of Prevalence of Azithromycin Resistance in S. aureus at Each Time Point” as requested
